# Creating inclusive libraries by applying universal design: a guide

**DOI:** 10.29173/jchla29669

**Published:** 2023-04-01

**Authors:** Catherine Boden

**Affiliations:** Assistant Dean, Research Support Services University of Saskatchewan, Saskatoon, Saskatchewan, Canada

Spina C. **Creating inclusive libraries by applying universal design: a guide**. Lanham: Rowman & Littlefield; 2021. Hardcover: 187p. ISBN: 9781538139776. Price: USD $95.00. Available from: https://rowman.com/ISBN/9781538139776/Creating-Inclusive-Libraries-by-Applying-Universal-Design-A-Guide

Carli Spina’s admirably readable *Creating inclusive libraries by applying universal design* provides librarians with a practical, theoretically grounded introduction to Universal Design (UD) and Universal Design for Learning (UDL). This book guides librarians in understanding and applying UD and UDL principles to make their products, spaces, and services more inclusive and welcoming. The following quote captures a key theme in this book: “To remain relevant when serving […] a diverse group of people with widely varied needs, libraries will need to focus on the type of flexibility, equity, and attention to user experience at the heart of Universal Design” [1].

UD is “an approach to design defined by its focus on the user and how it contemplates who the user is” (Spina)[1]. Historically, it was based in the need to design spaces to reduce barriers, particularly for individuals with disabilities. This book argues that the principles of Universal Design can be applied more broadly to reducing barriers to library spaces, services, and products, and making them welcoming to people with widely varied needs. Extending the concept of inclusion to the educational sphere, UDL is a framework that adapts UD “concepts and principles so as to make them meaningful in a broad range of educational settings and scenarios”[1].

Spina holds a JD from the University of Chicago Law School, an MLIS from Simmons GSLIS, and an MEd from the Harvard Graduate School of Education. She has contributed to work on equity, diversity, and inclusion in a variety of capacities, including serving in leadership roles on LITA’s Diversity and Inclusion Committee, and the ASCLA Library Services to People with Visual or Physical Disabilities that Prevent Them from Reading Standard Print Interest Group. Her experience and educational background are evident in the clarity of her writing, and the examples she employs to illustrate her points.

The first chapter sets the stage for the role of UD in libraries, with cautionary examples where accessibility was not at the forefront of the design process. Written essentially in two sections, this book then describes two related but distinct design philosophies, beginning with UD and moving on to UDL. For each design philosophy, the author:

- explores the history and context,
- defines the principles,
- examines limitations of the approach,
- describes the application of the design philosophy to libraries,
- provides illustrative case studies,
- and concludes with a practical checklist to assist the reader in applying the design principles.

The target audience is librarians working in any kind of library. While the book is grounded in the history of UD, with its roots in architecture and physical spaces, it swiftly moves to contextualize the application of UD and UDL in libraries. Spina’s extensive knowledge of these design philosophies, and their application in libraries, is evident in her writing. The notes and works cited at the end of each chapter provide the interested reader with the opportunity to pursue additional reading. The case studies illustrate the application of these design approaches in a diversity of library types, and checklists assist the reader in applying the principles in their own settings.

A frequent disappointment for me with practical guidebooks is an over-emphasis on positive attributes and a focus on convincing the reader about the merits of the approach to the detriment of a balanced presentation of strengths, weaknesses, and criticisms. No approach is without its limitations. A strength of this book is the inclusion of two chapters, chapters 4 and 10, on the limitations of UD and UDL respectively. These chapters bring balance to the reader’s understanding. As Spina notes, librarians need to understand the limitations of these design philosophies to make informed decisions of when and when not to apply them.

Readers should be aware that there tends to be a focus on the application of UD and UDL for physical and sensory disabilities. This is not a criticism of Spina’s writing but does reflect an area in need of development for UD and UDL. Quoting an essay by S.E. Smith, Spina highlights the gap between accessibility and true inclusion. She then notes that “ while Universal Design and UDL may not be sufficient to ensure these questions are fully answered in favor of true inclusion, a thoughtful application of their principles can serve as a starting point for this work”[1]. This is indeed how I interpret the use of these principles in my own work. I see UD and UDL as useful principles for accessibility and some aspects of inclusiveness in services and spaces, but not sufficient alone to make library services truly inclusive and welcoming. I was pleased the author acknowledged this limitation, and further, pointed to methods and resources outside the scope of the book to supplement UD and UDL principles.

The author works and lives in the United States and, understandably, references US laws and regulations. These sections of the book will be illustrative but less directly applicable to a Canadian audience.

As a title in the LITA guide series, this book imparts practical guidance in an easy to read format. It provides the reader with a strong grounding in the concepts and principles, practical advice and checklists in the use and application of UD and UDL. Though a slim book, there is a lot of content for the cost. I would recommend it for libraries or librarians wanting to make their services (particularly instruction) and spaces more accessible and welcoming to a wide diversity of individuals.
